# Psycho-Neuroendocrine-Immune Interactions in COVID-19: Potential Impacts on Mental Health

**DOI:** 10.3389/fimmu.2020.01170

**Published:** 2020-05-27

**Authors:** Ícaro Raony, Camila Saggioro de Figueiredo, Pablo Pandolfo, Elizabeth Giestal-de-Araujo, Priscilla Oliveira-Silva Bomfim, Wilson Savino

**Affiliations:** ^1^School of Medicine, Federal Fluminense University, Niterói, Brazil; ^2^Department of Neurobiology and Program of Neurosciences, Institute of Biology, Federal Fluminense University, Niterói, Brazil; ^3^National Institute of Science and Technology on Neuroimmunomodulation - INCT-NIM, Oswaldo Cruz Institute, Oswaldo Cruz Foundation, Rio de Janeiro, Brazil; ^4^Rio de Janeiro Research Network on Neuroinflammation, Oswaldo Cruz Institute, Oswaldo Cruz Foundation, Rio de Janeiro, Brazil; ^5^Laboratory on Thymus Research, Oswaldo Cruz Institute, Oswaldo Cruz Foundation, Rio de Janeiro, Brazil

**Keywords:** central nervous system, COVID-19, cytokine, HPA axis, mental health, pandemic, SARS-CoV-2, social isolation

## Abstract

Coronavirus disease 2019 (COVID-19) is caused by the Severe Acute Respiratory Syndrome Coronavirus 2 (SARS-CoV-2). The impacts of the disease may be beyond the respiratory system, also affecting mental health. Several factors may be involved in the association between COVID-19 and psychiatric outcomes, such as fear inherent in the pandemic, adverse effects of treatments, as well as financial stress, and social isolation. Herein we discuss the growing evidence suggesting that the relationship between SARS-CoV-2 and host may also trigger changes in brain and behavior. Based on the similarity of SARS-CoV-2 with other coronaviruses, it is conceivable that changes in endocrine and immune response in the periphery or in the central nervous system may be involved in the association between SARS-CoV-2 infection and impaired mental health. This is likely to be further enhanced, since millions of people worldwide are isolated in quarantine to minimize the transmission of SARS-CoV-2 and social isolation can also lead to neuroendocrine-immune changes. Accordingly, we highlight here the hypothesis that neuroendocrine-immune interactions may be involved in negative impacts of SARS-CoV-2 infection and social isolation on psychiatric issues.

## Introduction

In December 2019, a new outbreak of severe acute respiratory syndrome (SARS) emerged in Wuhan, China. Caused by Severe Acute Respiratory Syndrome Coronavirus 2 (SARS-CoV-2), the coronavirus disease 2019 (COVID-19) caused a national outbreak of severe pneumonia in China and quickly spread worldwide. According to the World Health Organization (WHO) official website, on May 6th, 2020, 3,595,662 people have been tested positive for SARS-CoV-2 infection and 247,652 deaths have resulted from SARS-CoV-2 worldwide (1). The disease, initially restricted to China, is now a pandemic, comprising all continents so far except for Antarctica, thus having become a major planetary health issue (1).

The most common symptoms of COVID-19 are fever, cough, dyspnea, sputum production, myalgia, headache, diarrhea, rhinorrhea, anosmia, and ageusia (2, 3). Nevertheless, symptoms of post-traumatic stress disorder (PTSD), anxiety and depression have also been prevalent in patients infected with COVID-19 (4, 5). Besides, SARS-CoV-2 RNA was detected in the cerebrospinal fluid of a patient (6) and increasing evidence points out that coronaviruses (CoVs) may invade the central nervous system (CNS) (7). Thus, we describe here the likely routes by which SARS-CoV-2 can invade the brain. Since COVID-19 is associated with increased levels of pro-inflammatory cytokines (8), an immune signature shared with several psychiatric disorders, we propose how the relationship between SARS-CoV-2/host can possibly impair interactions between the immune, nervous and endocrine systems, leading to psychiatric symptoms. Furthermore, once millions of people worldwide are isolated in quarantine to minimize the transmission of SARS-CoV-2 (9), we also discuss herein evidence on the negative impacts of social isolation measures upon mental health, gathering evidence that explains how social isolation can also lead to neuroendocrine-immune changes, impairing mental health. Accordingly, it is likely that both SARS-CoV-2 infection and social isolation epidemiological measures to contain the pandemic can lead to changes in psycho-neuroendocrine-immune circuits with impact on the appearance and/or evolution of mental health impairments in infected subjects, as well as in those individuals that, even though not being infected, are subjected to social isolation due to one or more risk factors. Finally, we provide some suggestions for how future research could confirm the hypotheses outlined here, as well as intervention strategies that mitigate the impact of COVID-19 pandemic on mental health.

## Neuroinvasive and Neuropathological Potential of SARS-CoV-2

Coronaviruses (CoVs) comprise a large enveloped non-segmented positive-sense RNA virus, which belong to the family *Coronaviridae*, within the order *Nidovirales* (10). They are classified in four genera, namely *Alphacoronavirus, Betacoronavirus, Gammacoronavirus*, and *Deltacoronavirus*, based on their phylogenetic relationships and genomic structures (10). The α-CoV and β-CoV are able to infect mammals, whereas the γ-CoV and δ-CoV tend to infect birds (11). Previously, six CoVs have been identified as capable of infecting humans (human coronaviruses—HCoVs): α-CoV HCoV-NL63 and HCoV-299E, and β-CoV HCoV-OC43, HCoVHKU1, Middle East Respiratory Syndrome Coronavirus (MERS-CoV), and Severe Acute Respiratory Syndrome Coronavirus (SARS-CoV). The last two HCoVs are considered the most lethal among them. However, the novel SARS-CoV-2 has shown a mortality rate that is presently also expressive (11).

SARS-CoV-2 infection leads to a clinical picture characterized by highly lethal pneumonia with symptoms similar to those reported for SARS-CoV and MERS-CoV (12). Genomic analysis show that SARS-CoV-2 shares highly homological sequence with SARS-CoV (13). Although the existence of more than one receptor for this virus cannot be excluded by now, evidence so far reveals that SARS-CoV-2 enters human host cells using the same receptor of SARS-CoV, the human angiotensin-converting enzyme 2 (hACE2) (14). Consequently, most of the infection mechanisms detailed for SARS-CoV could be applied to this novel virus.

HCoVs may enter the CNS through distinct routes: hematogenous and/or neuronal retrograde dissemination (7). The neuronal route can occur through at least two different pathways: (a) via olfactory nerves and/or (b) via enteric nervous system (7, 15). An experimental study using K18-*h*ACE2 transgenic mice for the expression of hACE2 (i.e., human SARS-CoV receptor) showed that SARS-CoV, when given nasally, could invade the brain, likely via the olfactory nerves (16). However, the non-expression of ACE2 in neurons in the olfactory system (17, 18) leads to question whether this is really a possible route for SARS-CoV-2 entry into CNS, although it is not yet possible to rule out the possibility that other ACE2-independent mechanisms are involved in the entry of SARS-CoV-2 into host cells. By contrast, ACE2 expression is abundant in small intestine endothelial cells (18), which connect with neurons in the enteric nervous system. In addition, gastrointestinal symptoms are commonly seen in a part of patients with COVID-19 (12, 19, 20) and SARS-CoV-2 was isolated from oral and anal swabs of these patients (21). In this way, the enteric nervous system, via the vagus nerve, can also be a possible pathway for SARS-CoV-2 to enter the CNS.

Similarly, the hematogenous route can occur by at least two mechanisms: (a) through infected leukocytes that cross the blood-brain barrier carrying the virus to the brain and/or (b) through direct infection of brain microvascular endothelial cells, which express ACE2 (22). Nonetheless, the hematogenous route does not seem to be involved in the CNS invasion by SARS-CoV, since virtually no viral particles were detected in non-neuronal cells of the infected brain areas in the early stage of infection (23–25). Yet, the precise route(s) by which SARS-CoV enters the CNS remain(s) to be determined. The recent SARS-CoV-2 RNA detection in the cerebrospinal fluid of a patient with COVID-19 (6), as well as its similarities with the SARS-CoV, emphasizes the need to conduct studies aiming at evaluating the neuroinvasive potential of SARS-CoV-2 in animal models and humans.

SARS-CoV genomic sequences in human brain tissues were found mainly in neurons of the cerebral cortex and hypothalamus, but not in the cerebellum (23, 24). However, pre-clinical studies with K18-*h*ACE2 mice infected by SARS-CoV revealed viral particles during acute phase in other brain regions besides the cortex and hypothalamus, such as cerebellum, midbrain (e.g., dorsal raphe and *substantia nigra*), thalamus, amygdala, hippocampus, basal ganglia (e.g., caudate-putamen and *nucleus accumbens*), cortex (e.g., frontal, infralimbic, and cingulate), and olfactory bulb (16, 26). In these animals, a rapid spread throughout the brain was accompanied by significant neuronal loss in the cingulate and infralimbic cortices and the anterior olfactory nucleus (26). Interestingly, high levels of cytokines and chemokines, most notably interleukin-6 (IL-6) and interferon gamma (INF-γ) were found in brain of K18-*h*ACE2 transgenic mice infected by SARS-CoV (16, 26). Rather surprisingly, minimal signals of local inflammation were observed, and apoptotic or necrotic cells were not detected (16).

Considering the high-expression of inflammatory mediators along with a lack of other inflammatory signals, how SARS-CoV can be leading to neuronal death remains unknown. Cell death non-inflammatory processes, such as autophagy, may be an explanation (16). Since autophagy is related to several neurodegenerative and psychiatric diseases (27), evaluating whether infection by SARS-CoV-2 can lead to neuronal death by autophagy may also be important for future relationships between SARS-CoV-2 infection and mental health outcomes.

## Impacts of the SARS-CoV-2 Infection on Mental Health

Several studies have demonstrated psychiatric manifestations in patients with MERS or SARS during the acute phase, such as increased stress levels, impaired memory, symptoms of depression, anxiety, PTSD, psychoses, and suicidal behavior (28–33). Long-term damage has also been seen in these patients. Survivors of SARS, months or years after the acute phase of the infection, may also exhibit impaired memory, sleep disturbances, increased levels of stress, depression, anxiety, and PTSD symptoms (32, 34–38). To date, few studies have evaluated the possible mental health outcomes of SARS-CoV-2 infection. However, corroborating the data observed in patients with SARS, a study recently demonstrated a prevalence of 96.2% of PTSD symptoms in 714 patients with COVID-19 during acute phase (4). Another study reported a prevalence of 34.72 and 28.47% of anxiety and depression symptoms, respectively, in 144 patients with COVID-19 (5). Taken together, these data indicate that infection with these HCoV, especially SARS-CoV-2, can yield a negative impact on mental health, both in the short- and long-term time windows. Supplementary Table 1 summarizes studies that reported mental health outcomes in patients with MERS, SARS, or COVID-19.

Many factors can influence the results of studies that have reported symptoms or development of psychiatric disorders in patients with MERS, SARS, or COVID-19. Among them (a) the work directly with health care, (b) the presence of family history of psychiatric illnesses, (c) less social support, (d) older age, (e) the isolation, and (f) the use of high doses of steroids during the acute phase (see Supplementary Table 1). However, some patients who survived SARS displayed psychiatric manifestations that appear to be disproportionate to the extent of lung infection or expected side effects of corticosteroid therapy (25, 28, 39). Furthermore, it has been reported that one patient developed progressive neurological symptoms starting at day 28 after the onset of the disease. This patient eventually died due to the SARS-CoV infection, and an autopsy revealed the presence of the virus in the brain, together with neuronal necrosis, glial hyperplasia, and edema (25). Although the studies cited above have been conducted with small samples of patients, they suggest that the psychiatric manifestations seen in at least some patients might be a direct effect of the infection of SARS-CoV. Also, studies with humans are important to evaluate and highlight the possible psychiatric outcomes in patients with SARS-CoV-2 infection.

### The Potential Role of Neuroimmune Network

A “cytokine storm” has been proposed as a key mechanism in the SARS-CoV-2 pathophysiology and related to lung damage and lethality observed in patients bearing COVID-19 (8). Accordingly, increased circulating levels of several cytokines have been found in patients with MERS, SARS, or COVID-19 (see Table 1). Interestingly, high levels of pro-inflammatory cytokines (e.g., IL-6 and INF-γ) were also found in the CNS of K18-*h*ACE2 transgenic mice infected by SARS-CoV (16, 26). This evidence supports the existence of an immune signature characterized by increased levels of pro-inflammatory cytokines involved in the pathophysiology of different pathogenic SARS-CoV in humans.

**Table 1:** Cytokines in blood of individuals with MERS, SARS, or COVID-19.

	**Sample size**	**Levels**	**References**
**MERS**			
IL-6	*n =* 9 severe vs. *n =* 8 mild *n =* 9 severe/fatal vs. *n =* 5 mild/moderate *n =* 24/30 infected	↑ ↑ ↑	(40) (41) (42)
TNF-α	*n =* 7 infected vs. *n =* 13 healthy	↑	(43)
IL-10	*n =* 9 severe/fatal vs. *n =* 5 mild/moderate	↑	(41)
INF-γ	*n =* 7 infected vs. *n =* 13 healthy	↑	(43)
IFN-α	*n =* 9 severe vs. *n =* 8 mild	↑	(40)
IL-2	*n =* 7 infected vs. *n =* 13 healthy	−	(43)
IL-12	*n =* 7 infected vs. *n =* 13 healthy	−	(43)
IL-13	*n =* 7 infected vs. *n =* 13 healthy	−	(43)
IL-4	*n =* 7 infected vs. *n =* 13 healthy	−	(43)
IL-15	*n =* 7 infected vs. *n =* 13 healthy	↑	(43)
IL-17	*n =* 7 infected vs. *n =* 13 healthy	↑	(43)
**SARS**			
IL-6	*n =* 14 infected vs. *n =* 12 healthy *n =* 20/20 infected *n =* 30 severe > *n =* 30 mild/moderate > *n =* 30 convalescent/*n =* 20 healthy *n =* 14/14 infected *n =* 23 infected vs. *n =* 25 healthy *n =* 88 infected vs. *n =* 10 healthy *n =* 61 infection initial stage vs. *n =* 44 healthy	↑ ↑ ↑ − ↑ ↑ −	(44) (45) (46) (47) (48) (49) (50)
IL-1β	*n =* 20/20 infected *n =* 14/14 infected	↑−	(45) (47)
TNF-α	*n =* 14 infected vs. *n =* 12 healthy *n =* 20/20 infected *n =* 30 severe vs. *n =* 30 mild/moderate vs. *n =* 30 convalescent vs. *n =* 20 healthy *n =* 8 dead infected vs. *n =* 6 survivors infected *n =* 61 infected vs. *n =* 44 healthy *n =* 24 infected vs. *n =* 12 healthy	− − − ↑ ↑ ↑	(44) (45) (46) (47) (50) (51)
IL-10	*n =* 14 infected vs. *n =* 12 healthy *n =* 88 infected vs. *n =* 10 healthy	− −	(44) (49)
IFN-γ	*n =* 14 infected vs. *n =* 12 healthy *n =* 20/20 infected *n =* 88 infected vs. *n =* 10 healthy	− ↑ ↑	(44) (45) (49)
IL-2	*n =* 14 infected vs. *n =* 12 healthy	↑	(44)
IL-12	*n =* 20/20 infected	↑	(45)
IL-8	*n =* 14/14 infected *n =* 14 infected vs. *n =* 12 healthy *n =* 14 infected vs. *n =* 12 healthy *n =* 30 severe/*n =* 30 mild/moderate vs. *n =* 20 healthy *n =* 23 infected vs. *n =* 25 healthy *n =* 88 infected vs. *n =* 5 healthy *n =* 18 infected vs. *n =* 12 healthy	− − ↑ ↓ ↑ − ↑	(47) (44) (44) (46) (48) (49) (51)
IL-16	*n =* 61 infected vs. *n =* 44 healthy	↑	(50)
IL-13	*n =* 61 infection initial stage vs. *n =* 44 healthy	↑	(50)
TGF-β	*n =* 30 severe/*n =* 30 mild/moderate vs. *n =* 20 healthy *n =* 66 infected vs. *n =* 5 healthy *n =* 61 infected vs. *n =* 44 healthy	↓ − ↑	(46) (49) (50)
IL-4	*n =* 14 infected vs. *n =* 12 healthy	−	(44)
**COVID-19**			
IL-6	*n =* 13 ICU vs. *n =* 4 healthy *n =* 286 severe vs. *n =* 166 moderate *n =* 5 critical > *n =* 9 severe > *n =* 5 mild *n =* 2/8 ICU *n =* 15 severe vs. *n =* 28 mild *n =* 69 severe vs. *n =* 11 non-severe *n =* 11 severe vs. *n =* 10 moderate *n =* 7 SpO_2_ <90% vs. *n =* 36 SpO_2_≥90%	↑ ↑ ↑ ↑ ↑ ↑ ↑ ↑	(12) (52) (53) (54) (55) (56) (57) (58)
IL-1β	*n =* 41 infected vs. *n =* 4 healthy *n =* 11 severe vs. *n =* 10 moderate	↑ ND	(12) (57)
TNF-α	*n =* 41 infected vs. *n =* 4 healthy *n =* 13 ICU vs. *n =* 28 non-ICU *n =* 286 severe vs. *n =* 166 moderate *n =* 5 critical vs. *n =* 9 severe vs. *n =* 5 mild *n =* 69 severe vs. *n =* 11 non-severe *n =* 11 severe vs. *n =* 10 moderate	↑ ↑ ↑ − − ↑	(12) (12) (52) (53) (56) (57)
IL-10	*n =* 41 infected vs. *n =* 4 healthy *n =* 13 ICU vs. *n =* 28 non-ICU *n =* 286 severe vs. *n =* 166 moderate *n =* 5 critical vs. *n =* 9 severe vs. *n =* 5 mild *n =* 5/8 ICU *n =* 69 severe vs. *n =* 11 non-severe *n =* 11 severe vs. *n =* 10 moderate *n =* 7 SpO_2_ <90% vs. *n =* 36 SpO_2_≥90%	↑ ↑ ↑ − ↑ − ↑ ↑	(12) (12) (52) (53) (54) (56) (57) (58)
IFN-γ	*n =* 41 infected vs. *n =* 4 healthy *n =* 2/8 ICU *n =* 69 severe vs. *n =* 11 non-severe	↑ ↑ −	(12) (54) (56)
IL-2	*n =* 13 ICU vs. *n =* 4 healthy *n =* 13 ICU vs. *n =* 28 non-ICU *n =* 69 severe vs. *n =* 11 non-severe	↑ ↑ −	(12) (12) (56)
IL-2R	*n =* 286 severe vs. *n =* 166 moderate *n =* 5 critical > *n =* 9 severe > *n =* 5 mild *n =* 11 severe vs. *n =* 10 moderate	↑ ↑ ↑	(52) (53) (57)
IL-4	*n =* 69 severe vs. *n =* 11 non-severe	−	(56)

*(↑), increase; (↓), decrease; (−), no changes; ND, not detectable*.

*COVID-19, Coronavirus Disease 2019; SARS, Severe Acute Respiratory Syndrome; MERS, Middle East Respiratory Syndrome; ICU, Intensive Care Unit; SpO_2_, peripheral capillary oxygen saturation; IL, Interleukin; TNF-α, Tumor Necrosis Factor alfa; TNFR, Tumor Necrosis Factor Receptor; IFN-γ, Interferon gamma; TGF-β, Transforming Growth Factor beta; IL-2R, Interleukin-2 Receptor*.

Furthermore, higher serum levels of pro-inflammatory cytokines (e.g., IL-6 and IFN-γ) and chemokines were found in SARS patients with severe disease, as compared to individuals with uncomplicated SARS (44–46). Recently, dysregulation of the immune response similar to SARS-CoV infection has been observed in patients with SARS-CoV-2 in Wuhan (China). Particularly, a significant increase in the serum levels of several pro-inflammatory cytokines, or corresponding cytokine receptors, in severe patients (*n* = 286) than the non-severe ones (*n* = 166), including IL-6, tumor necrosis factor alpha (TNF-α) and interleukin-2 receptor (IL-2R) (52). Similarly, intensive care unit (ICU) patients (*n* = 13) with severe SARS-CoV-2 infection displayed higher plasma levels of cytokines, such as IL-2 and TNF-α, when compared with non-ICU patients (*n* = 28) (12).

A previous study identified psychiatric manifestations (e.g., psychosis, cognitive impairments, depression, and anxiety symptoms) in patients during the acute phase of SARS-CoV infection (28). The authors also found an association between the severity of symptoms and some psychiatric outcomes. If the increase in cytokine levels and the manifestation of psychiatric symptoms are related to the severity of the symptoms of SARS-CoV infection, the “cytokine storm” might also be related to the “mental health thunderstorms” seen in patients with COVID-19?

Accordingly, a possible mechanism concerning the relationship between SARS-CoV-2 infection and mental health outcomes is the involvement of neuroimmune networks. Table 2 shows that increased levels of various cytokines can be seen in several psychiatric disorders, an immune signature shared with the SARS-CoV-2 infection. Soluble cytokines that reach the brain, or corresponding local altered levels can influence synthesis, release and reuptake of several neurotransmitters, including monoamines, such as dopamine, norepinephrine, and serotonin (78). Changes in the metabolism of neurotransmitters are involved in the pathophysiology of various psychiatric disorders, such as depression, anxiety, PTSD, and obsessive-compulsive disorder (79, 80). Since changes in cytokine levels can lead to a disruption in the metabolism of neurotransmitters, triggering behavioral deficits, we hypothesize than the immune system can be placed as a link between SARS-CoV or SARS-CoV-2 infection and mental health impairments.

**Table 2:** Increased levels of cytokines in psychiatric disorders (data based on meta-analyzes).

**Cytokines**	**Psychiatric disorders**	**Tissue**	**References**
IL-6	Depressive disorders Schizophrenia PTSD Sleep disorder	Blood/CSF Blood/CSF Blood Blood	(59) (60) (61) (62) (63) (64) (65) (66) (67) (68) (69) (65) (70) (71) (72)
IL-6R	Bipolar disorder	Blood	(73) (74)
IL-1β	Depressive disorders Schizophrenia Bipolar disorder PTSD	Blood CSF CSF Blood	(66) (65) (65) (70) (71)
IL-1RA	Depressive disorders Schizophrenia	Blood Blood	(64) (75) (68)
TNF-α	Depressive disorders Bipolar disorder PTSD	Blood/post-mortem brain Blood Blood	(60) (64) (59) (63) (67) (74) (70) (71)
TNFR-1	Bipolar disorder	Blood	(73) (74)
TNFR-2	Depressive disorders	Blood	(64)
IL-10	Depressive disorders Suicide	Blood Blood	(64) (76)
IFN-γ	PTSD	Blood	(70) (71)
IL-2	PTSD	Blood	(71)
IL-2R	Depressive disorders Bipolar disorder Schizophrenia	Blood Blood Blood	(60) (64) (73) (74) (69) (68)
IL-12	Depressive disorders Schizophrenia	Blood Blood	(64) (75)
IL-13	Depressive disorders	Blood	(64)
IL-18	Depressive disorders	Blood	(64)
IL-8	Schizophrenia	CSF	(65)
IL-4	Bipolar disorder	Blood	(73) (74)
TGF-β	Suicide	Blood	(77) (76)

*IL, Interleukin; IL-R, Interleukin Receptor; TNF-α, Tumor Necrosis Factor alfa; TNFR, Tumor Necrosis Factor Receptor; IFN-γ, Interferon gamma; TGF-β, Transforming Growth Factor beta; PTSD, Post-traumatic stress disorder; CSF, cerebrospinal fluid*.

Evidence shows that cytokines also play a key role in learning and memory processes. In healthy conditions, an increase in gene expression of IL-1β, IL-1 receptor antagonist, IL-6, and IL-18 occurs in hippocampus during long term potentiation (LTP), a process considered to underlie certain forms of learning and memory (81–83). While IL-1β is related to LTP maintenance, acquisition of learning and memory consolidation, IL-6 has opposite effects. However, during peripheral and central diseases in which the brain levels of IL-1β and IL-6 are increased, both cytokines tend to inhibit the synaptic plasticity, learning, and memory (84). Importantly, high levels of IL-6 were found in blood of SARS-CoV and SARS-CoV-2 infected patients (see Table 1), as well as in CNS of K18-*h*ACE2 transgenic mice infected by SARS-CoV (16, 26). Impaired memory has also been observed in both acute and convalescent phases of SARS infection in humans (see Supplementary Table 1). Therefore, it is possible that the increased levels of IL-6 are related to the cognitive impairments observed in SARS patients. Such issue should be evaluated in future studies.

Interleukin-6 is a well-known pleiotropic cytokine expressed in low levels in healthy individuals, in the presence of homeostasis alterations it becomes higher and rapidly detected, and even after stress agent removal, its levels can be maintained elevated and cause diseases (81, 85). Accordingly, a dysregulation of this cytokine expression counts for the development of psychiatric disorders (86), as seen in Table 2.

Recently, Gao et al. (55) showed increased levels of cytokines in patients with SARS-COV-2, especially IL-6, which seems to be directly related to the severity of the disease. Evaluating the blood parameters of 43 adult patients positive to SARS-CoV-2 and subdivided in groups (mild and severe) they found a significant increase in the combined detection of IL-6 and D-dimer specially in the severe cases, pointing out the IL-6 and D-dimer combination as a potential biomarker to identify early stages or the prognosis of the COVID-19 disease (55). In another study, 29 patients were subdivided in three groups (mild, severe, and critical) and had hematological parameters followed up during disease evolution. It was shown that the more severe the case was, the higher was the IL-6 level (53). Liu et al. demonstrated that not only increased levels of IL-6 related to the severity of COVID-19, but also that decreased levels of IL-6 were positively correlated with the treatment effectiveness and remission of the disease (56).

In this sense, the humanized anti-interleukin-6-receptor (IL-6R) monoclonal antibody (Tocilizumab), a drug used against rheumatoid arthritis (85) that inhibits IL-6 signaling, has been administered experimentally in treatment of COVID-19 (87). The retrospective evaluation of 21 patients demonstrated that Tocilizumab was able to improve the respiratory function and restored the levels of lymphocytes in the blood, which can be promising (87). In a second vein, a meta-analysis study pointed out that treatment with anti-cytokine drugs, including Tocilizumab, may have an antidepressant effect (88). Accordingly, we can conceive that this type of treatment may represent a promising therapeutic alternative to be attempted in humans, not only has beneficial effects for respiratory symptoms associated with COVID-19, but also for possible depressive symptoms related to the disease. Thus, it would be interesting for future clinical studies to evaluate the effects of Tocilizumab and other pharmacological treatments not only on symptoms and tests related to respiratory and immune functions, but also on the psychiatric symptoms.

It is important to notice that some individual biological characteristics associated with impaired immunity may influence not only the natural history of COVID-19, but also the associated psychiatric outcomes. In this context, obesity, which is linked with systemic inflammation and impaired immunity, can increase vulnerability for COVID-19 (89, 90), contributes to neuroinflammation and constitutes an important risk factor for the development or worsening of psychiatric disorders [for review, see (91)]. Another important factor is aging, which is related to an imbalance in the levels of pro-inflammatory (high levels) and anti-inflammatory (low levels) cytokines and decrease in T-cell-mediated function (92). These immunosenescence-dependent changes in the elderly may be associated with higher susceptibility to viral diseases, including COVID-19 (93), as well as neuropsychiatric disturbances, such as cognitive impairments (94). It has been demonstrated the relationship between aging and symptoms of anxiety and depression in patients infected with SARS-CoV-2 during the acute phase (5). Therefore, both obesity and older age may increase the risk of psychiatric symptoms in patients with COVID-19; and one hypothesis is that neuroimmune circuits may be involved in this association. In addition, since poor nutrition and sedentary lifestyle are frequent in the elderly population and in overfat individuals, actions that promote the practice of physical activity and adequate nutrition are crucial, as they can potentially be associated with a lower risk for COVID-19 and mental health impairments.

Pregnancy is another important potential factor that can affect the neuropsychiatric outcomes of COVID-19. Maternal immune activation (e.g., in response to infection) is a risk factor for neurodevelopmental disorders such as autism spectrum disorder (ASD) (95). Autism has a complex etiology, involving environmental, and genetic factors. One of the proposed etiologies for ASD is viral infection in early stages of development (96). Although the mechanisms by which viral infection can lead to autism are not yet known, it is believed that they may occur through (a) direct infection of the infant CNS, or (b) due to the inflammatory response of the mother and/or the fetus, which can lead to neuroinflammation, triggering changes in brain development (96). In fact, clinical evidence supports the participation of the neuro-immune mechanisms in the pathophysiology of ASD [for review, see (97)]. While increasing evidence supports the neuroinvasive potential of SARS-CoV-2, there is still no consistent demonstration of vertical transmission of this virus. In this sense, a recent study reviewing the effects of SARS, MERS, and COVID-19 on gestational outcomes, including vertical transmission, and demonstrated that fortunately this transmission mechanism does not appear to occur in these betacoronaviruses (98). However, the controversial data on this aspect and the high expression of ACE2 detected in the human placenta (99) revealed that the possibility of vertical transmission needs to be further explored in clinical settings. Accordingly, it is important to point out that there is still insufficient evidence to support the association between SARS-CoV-2 infection during pregnancy and the development of ASD. Nonetheless, we cannot rule out that changes in the maternal immune response triggered by the SARS-CoV-2 infection may affect neurodevelopment, another aspect that also deserves the attention of the medical and scientific communities.

In any case, since increased levels of cytokines have been observed in COVID-19 and in psychiatric disorders, it is likely that changes in neuroimmune axes may be involved in the mental health outcomes occurring in COVID-19 patients. Although this hypothesis is based mainly on studies with other beta-coronaviruses, it will be interesting if future clinical studies, for example, include the search for correlations between the levels of inflammatory markers and psychiatric symptoms in COVID-19 patients and survivors. Studies in animal models infected with SARS-CoV-2 may also assist in the investigation of possible pathological mechanisms involved in neurobehavioral disorders related to the viral infection.

### The Potential Role of Neuroendocrine-Immune Axes

**Figure 1:**
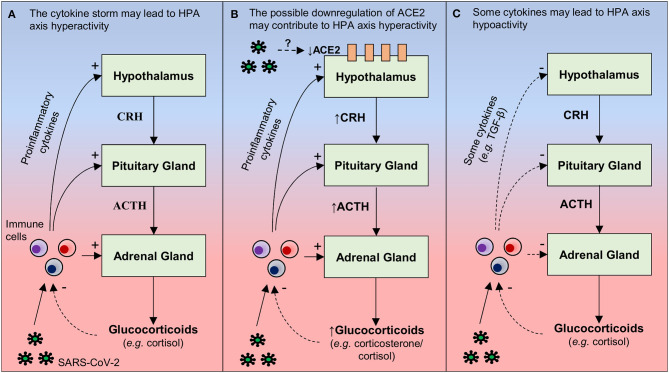
Hypothetical mechanisms by which SARS-CoV-2 may lead to changes in the activity of the hypothalamus-pituitary-adrenal (HPA). **(A)** During a viral infection (e.g., SARS-CoV-2), pro-inflammatory cytokines are released by immune cells present in the periphery (e.g., macrophages, T and NK cells) and/or in the brain (microglia). These cytokines can act at three levels of the HPA axis: increasing (i) the secretion of the corticotrophin-releasing hormone (CRH) in the hypothalamus, (ii) the secretion of adrenocorticotropic hormone (ACTH) in the pituitary, and (iii) release of glucocorticoids (e.g., cortisol) through the adrenal cortex. By any of these actions, the result is an increased release of glucocorticoids, which bind to their receptors present in immune cells, suppressing the synthesis and release of pro-inflammatory cytokines. Therefore, it is possible that increased pro-inflammatory cytokine levels in COVID-19 may lead to hyperactivity of the HPA axis. However, due to a dysfunction in the negative feedback between the HPA axis and the immune system, this neuroendocrine axis is not able to reduce the production of inflammatory mediators, a possible explanation for why SARS-CoV-2 infection leads to cytokine storm. **(B)** Hypothalamic ACE2 overexpression decreases the activity of the HPA axis in mice, reducing the CRH content in the hypothalamus and corticosterone plasma levels. Since SARS-CoV infection is able to reduce the expression of ACE2 in other tissues, one hypothesis (based on molecular similarities between SARS-CoV-2 and SARS-CoV) is that SARS-CoV- 2 can induce a decrease in hypothalamic ACE2 levels, thus contributing to HPA hyperactivity. **(C)** Although pro-inflammatory cytokines classically increase the activity of the HPA axis, some cytokines (e.g., TGF-β) can decrease the activity of this neuroendocrine axis under specific conditions that remain unclear. This is another mechanism by which the SARS-CoV-2 infection, inducing an exacerbated inflammatory response, may lead to changes in the HPA axis, in this case, hypoactivity. Continuous arrows: stimulation; dashed arrows: inhibition.

The activation of the hypothalamic-pituitary-adrenocortical (HPA) axis has been observed during pathologies involving an immune/inflammatory process, including viral infections (100). The activation of this neuroendocrine axis by pro-inflammatory cytokines causes increased glucocorticoid production, a physiological response that contributes to avoid the deleterious effects of excessive production of inflammatory mediators and a non-specific recruitment of cells with no or low affinity for triggering antigens (101). In this respect, it seems reasonable to imagine a state hyperactivity of the HPA axis in infected patients, due to the “cytokine storm” observed in these individuals (Figure 1A).

A second aspect deserving discussion is the fact that ACE2 overexpression in corticotropin-releasing-hormone (CRH)-producing neurons in the hypothalamic paraventricular nucleus alters the processing of psychogenic stress in mice, decreasing the CRH content in the hypothalamus and corticosterone plasma levels (i.e., less HPA axis activation), as well as anxiety-like behaviors (102). SARS-CoV infection decreases the expression of ACE2 in the lungs and myocardium of infected mice (103, 104). Also, patients who died from SARS and had SARS-CoV detected in the hearts exhibited reduced ACE2 levels, when compared to patients who died from a non-SARS related sepsis (104). Although SARS-CoV genomic sequences have been found in the hypothalamus of humans (24), it remains to be determined whether the virus also decreases ACE2 contents in this brain region. In any case, a downregulation of hypothalamic ACE2 levels may be considered as another potential mechanism by which SARS-CoV/SARS-CoV-2 induces hyperactivity of the HPA axis with consequent psychiatric disturbances that are observed in these patients, such as the anxiety for example (Figure 1B). However, the role of ACE2 in the SARS-CoV-2 pathogenesis is still unknown and more studies are needed to test this mechanism.

By contrast, in a study that prospectively assessed the presence of hormonal changes in 61 SARS survivors (without pre-existing endocrine disorders) 3 months following recovery, 24 patients (39.3%) displayed late HPA axis hypoactivity, with hypocortisolism (105). This alteration appeared to be a pathological effect of SARS-CoV, since nearly two-third of the patients did not use steroids and the majority were young (mean age: 36.5 years) and previously healthy (105). Retrospective data from SARS survivors do not support changes in HPA axis activity during the acute phase, suggesting that SARS-associated hypocortisolism is a late onset phenomenon (105). Since the “cytokine storm” is seen in the acute phase of SARS (see Table 1), increased cytokine levels are unlikely to be secondary to HPA axis hypofunction. Although pro-inflammatory cytokines classically increase the activity of the HPA axis (i.e., a downregulation mechanism of the inflammatory response), under some conditions, TNF-α and transforming growth factor beta (TGF-β) may induce HPA axis hypoactivity (106). Therefore, it is possible that some cytokines that are increased in SARS patients play a causative role in SARS-associated hypocortisolism. As both hyperactivity and hypoactivity of the HPA axis are associated with depression (107, 108), hypocortisolism can also be associated with depressive symptoms that can be in SARS survivors. In addition, due to the similarities between SARS-CoV-2 and SARS-CoV, it is possible that this mechanism involved in HPA axis hypoactivity can also be observed in COVID-19 (Figure 1C). Thus, studies that simultaneously evaluate the axis HPA activity, cytokine levels, and psychiatric disturbances in patients and survivors of COVID-19 will certainly improve the current knowledge.

In the above context, it is noticeable that long-term survivors of the acute respiratory distress syndrome often report traumatic memories from the ICU. Interestingly, these patients displayed lower baseline cortisol levels and higher incidence of PTSD (109). Such an information leads to questions related to the hypocortisolism observed in SARS-CoV infected patients, which may reflect an exhaustion of the adrenal cortex function, as a result of the viral infection or distress associated with hospitalization. Clearly, future studies are needed to assess whether SARS-CoV-2 can affect the functioning of the HPA axis and whether this is involved in the association between SARS-CoV-2 infection and mental health outcomes. In clinical settings, it will be important to observe and measure the stress associated with hospitalization, as well as the presence of traumatic memories, as these factors may also be associated with changes in the HPA axis.

A dysfunctional glucocorticoid-immune circuitry has been observed in schizophrenia. After a stress paradigm, while healthy patients experienced an increase in cortisol levels, negatively correlated to the subsequent changes in IL-6 levels, patients with schizophrenia had elevated cortisol positively correlated to subsequent changes in IL-6 levels, suggesting an inability to down-regulate inflammatory responses to psychological stress in this psychiatric condition (110). It is well-known that stressful life events may precipitate subsequent exacerbations of the illness (111). Interestingly, elevated levels of circulating IL-6 have been found in early episode psychosis patients (112). Increased levels of stress or IL-6 have also been described in SARS or COVID-19 patients (see Supplementary Table 1 and Table 1). In addition, several studies reported symptoms of psychosis during the acute or long-term phase in SARS patients (see Supplementary Table 1). Therefore, it is possible that SARS-CoV-2 infection and stressors related to hospitalization may increase the risk of psychosis by increasing levels of cytokines and/or by disrupting the glucocorticoid-immune circuits. Since infections are associated with increased risk of developing schizophrenia (113), it seems important that future studies further assess the potential association between SARS or COVID-19 and the development of schizophrenia, as well as highlighting the importance of measures that prevent or reduce the impact of COVID-19 on mental health.

Therefore, it is possible that increased pro-inflammatory cytokine levels in COVID-19 lead to hypoactivity or hyperactivity of the HPA axis and, due to a dysfunction in the negative feedback between the HPA axis and the immune system, this neuroendocrine axis is not able to reduce the production of inflammatory mediators. In this sense, we hypothesize that such a dysfunction in the negative feedback between the HPA axis and production of pro-inflammatory cytokines may also be associated with mental health outcomes of the SARS-CoV-2 infection, thus conceptually corresponding to a psycho-neuroendocrine-immune dysfunction. Pre-clinical studies will hopefully provide more consistent clues to define a putative causal association between SARS-CoV/SARS-CoV-2 infection and behavioral deficits. In addition, animal models should allow a better control of variables that could also affect this association, such as the isolation of infected patients, since social isolation *per se* can also lead to both immunological and behavioral dysfunctions. In the current scenario, where social isolation measures are being strongly implemented worldwide, it is also important put into focus the potential damage to the mental health of isolated individuals, infected or not, applied the psycho-neuroendocrine-immune approach discussed herein.

## Impacts of Social Isolation on Mental Health

The exponential increase in the number of people infected with SARS-CoV-2 is leading to saturation of health services worldwide. To prevent human-to-human transmission and, in this way, slow down the growth of the pandemic, WHO has recommended that people avoid getting outside as much as possible (9). Although such a measure is necessary to contain the advance of the pandemic, social isolation can cause negative impacts on mental health of individuals.

Studies on mental health outcomes of the quarantine during other epidemics, including SARS and MERS, revealed negative psychological effects, such as symptoms of PTSD, depression, stress, anxiety, and fear. Some of the predictors of psychological impact included having a history of psychiatric illness, health-care work, longer quarantine duration, infection fears, boredom, inadequate supplies, inadequate information, and financial resources (114).

Results of an online survey that assessed the levels of psychological impact and stress during the initial stage of COVID-19 outbreak were recently reported (115). The responses of 1,210 subjects showed that 8.1, 28.8, and 16.5% had moderate to severe stress levels, anxiety and depression symptoms, respectively. Moreover, the general public with no formal education had a significant greater likelihood of depression during epidemic and higher satisfaction with the health information received was associated with a lower mental health impact of outbreak. People that presented SARS-CoV-2-related symptoms like coryza, cough, dizziness, and myalgia or reported a history of chronic illnesses showed significant high levels of anxiety, depression, and stress. These results suggest an importance of accurate health information to reduce the impact of rumors and show the need for the media to provide, not only true information, but also information in simple language so that to support those people with less educational background during the epidemic (115). In addition, these data lead to the urgent need of psychological and psychiatric interventions, together with measures to prevent the spread of SARS-CoV-2, so that to provide, as much as possible, well-being to both infected and non-infected socially isolated people.

Several studies show that living alone (vs. living with a family member) is associated with elevated levels of depressive symptoms (116–118), higher risk of depression (119), and higher mortality (120). Yet, it has been emphasized the need for caution in arguing for a negative association between living alone and mental health (121). One reason is that other factors may influence the association between living arrangements and mental health, such as social networks (121, 122), social support (116, 123) and neighborhood environment (117, 118, 124). In a study using data from more than 20,500 individuals in the United Kingdom or England, it was shown that prevalence of common mental disorders was higher in people living alone vs. people not living alone. This association occurred regardless of age and gender but was largely mediated by loneliness. Therefore, we believe that people living alone may be more vulnerable to the effects of quarantine on mental health than people living with a family member. Accordingly, it would be interesting for future studies to assess the influence of different living arrangements on outcomes of quarantine on mental health.

In this framework, loneliness has been associated with several psychiatric disorders, such as depression, anxiety, and suicide behavior (125). Importantly, it has been showed that lonely people present several immune dysregulations, such as upregulated expression of pro-inflammatory cytokine genes (126). On the other hand, several studies have revealed that changes in the immune system play a key role in mental disorders (127). Therefore, it is possible that changes in the immune system are involved in the negative impacts of loneliness on mental health. Accordingly, it is conceivable that inflammatory mediators are also involved in the impact of quarantine on mental health, during COVID-19.

Studies with animal models have provided important clues on the neurobiological and the behavioral consequences of social isolation. In rodents, the stress of social isolation is able to lead to changes in several neurotransmitter systems (e.g., dopaminergic, adrenergic, serotonergic, gabaergic, glutamatergic, nitrergic, and opioid systems). Indeed, the synthesis, release and even the corresponding receptor expression can be altered in several brain regions (e.g., hippocampus, cortex) of animals submitted to social isolation stress [for review, see (128)]. Disturbances in neuroplasticity-related signaling pathways are also observed in these models (128). For instance, rats submitted to chronic social isolation stress displayed brain morphological changes such as decreased number of dendritic spines in the hippocampus and prefrontal cortex, as well as decreased brain-derived neurotrophic factor (BDNF) and phosphorylated-protein kinase B (p-Akt) in the dorsal hippocampus (129). The BDNF/TrkB/PI3K/Akt pathway had already been described to be an important pathway in the maintenance of synaptic plasticity through translation and transport of synaptic proteins (130, 131). In this context, a metanalysis study reported a positive correlation between lower BDNF serum levels and depressive symptoms (132), and patients who present depressive symptoms may have reduced hippocampal volume (133), which supports the association between neuroplasticity and depressive disorders.

The social isolation stress can also lead to hyperactivity of the HPA axis through an increase in corticosterone production and release in rodents (134). The abnormal levels of glucocorticoid have been related to depressive-like behavior and can affect the hippocampal neurogenesis (135). Additionally, social isolation stress can lead to neuroinflammation, with higher levels of toll-like receptors, IL-6 and TNF-α in the hippocampus (136), as well as increased plasma levels of TNF-α, IL-4, IL-10, and ACTH in isolated rats (137). A recent systematic review reported that social isolation and loneliness may be linked to systemic inflammation (i.e., high levels of C-reactive protein and IL-6) in the general population (138). Accordingly, it is conceivable that nervous, immune and endocrine systems can be interacting with each other, mediating neurobehavior impairments induced by social isolation stress. Thus, these interactions may be part of the mechanisms by which social isolation during quarantine, via changes in neuroendocrine-immune circuits, can trigger damage to mental health. Yet, future studies are needed to understand the mechanisms associated with the psychological damage caused by quarantine.

Although the whole population can be affected by the psychological impacts of COVID-19, some vulnerable groups may experience the same pandemic scenario differently. A recent study based on a multidisciplinary approach called attention for measures that can support the population susceptibilities such as (1) older adults with multicomorbidities, (2) children and women that stay at home and suffer domestic violence, (3) people with preexisting mental health issues, (4) people with learning difficulties, which might be affected by disruption to support and by loneliness, (5) front-line health care workers that can be affected by the fear of infection, and (6) groups that have hard socio-economic difficulties (22).

As previously mentioned, financial problems may enhance the impact of social isolation on mental health during quarantine (114). Interestingly, studies demonstrated that a worse socioeconomic status is directly related to higher systemic levels of inflammatory markers such as IL-6 and C-reactive protein [for review, see (139)]. Thus, it is possible that neuroimmune interactions may also be involved in the impacts of financial stress during COVID-19 on mental health. This represents a novel possibility, that for sure requires future investigation. In addition, higher levels of inflammatory markers associated with worse socioeconomic conditions may also explain why lower social support is also associated with symptoms of anxiety and depression in patients infected with SARS-CoV-2 (5). Even though the biological mechanisms involved in the impact of socioeconomic status on mental health are still unclear, actions aiming at reducing socioeconomic inequalities should be a priority, in order to mitigate the impacts of COVID-19 on mental health.

Finally, it is important to note that the evidence highlighted here does not contradict the need for the isolation measures that are necessary to control the pandemic. However, they call attention to the usefulness of strategies aiming at reducing the harmful effects of social isolation on mental health of the general public, including the improvement of psychological intervention and the reduction of socioeconomic inequalities.

## Discussion

In summary, previous studies have reported psychiatric manifestations in patients infected with SARS-CoV-2, such as anxiety, depression and PTSD symptoms (4, 5). Since increased levels of cytokines have been observed in COVID-19 and in psychiatric disorders, we can place immune/inflammatory pathways as one of the mechanisms involved in mental health outcomes of COVID-19. Changes in the HPA axis have also been observed in SARS patients, indicating that alterations in neuroendocrine-immune circuits may be related to the psychiatric symptoms observed in these individuals. Therefore, the hypothesis of the present article is that SARS-CoV-2 infection can lead to neuroinflammatory and endocrine changes, which in turn may reflect poor mental health. However, it is important to note that related biological factors (e.g., older age, female gender, and overfat), together with other factors inherent to COVID-19 (e.g., social isolation, financial stress, and adverse effects of treatments) can influence psychiatric outcomes. Accordingly, it is likely that the psychiatric symptoms observed in COVID-19 patients are due to processes involved in the virus-host relationship, as well as to psychosocial and therapeutic issues associated with the pandemic.

**Figure 2:**
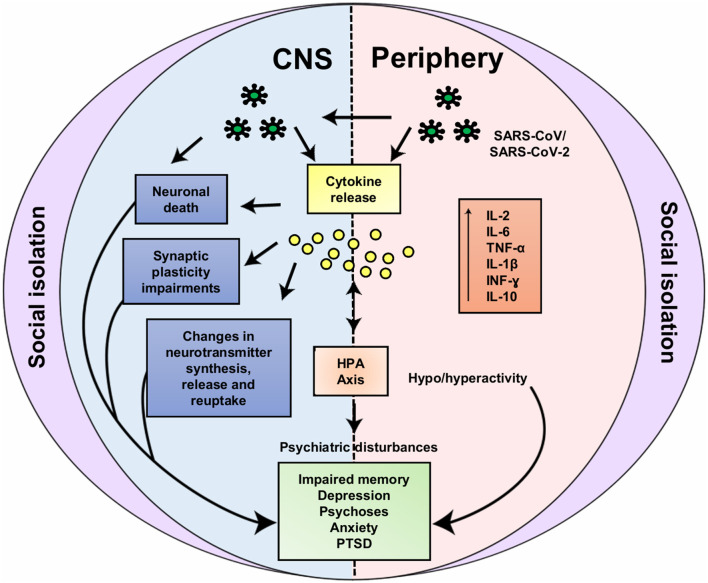
Possible neuroendocrine-immune interactions involved in impacts of SARS-CoV-2 infection and social isolation on mental health. Based on the similarity of SARS-CoV-2 and SARS-CoV, hematogenic or neuronal retrograde dissemination routes (via olfactory nerve) may be involved in the entry of the SARS-CoV-2 into the central nervous system (CNS). In the CNS **(left)** the virus can lead to increase in cytokines levels (e.g., IL-2, IL-6, TNF-α, IL-1β, INF-γ, and IL-10) due to its local or peripheral **(right)** actions. Increased cytokine levels are associated to neuronal death, synaptic plasticity impairments, dysfunction in the neurotransmitter metabolism and in the hypothalamic-pituitary-adrenocortical (HPA) axis. Likewise, social isolation can also lead to these neuroendocrine-immune disturbances, for instance: increase in cytokine levels, changes in neurotransmitter systems, HPA axis hyperactivity and disturbances in neuroplasticity-related signaling pathways. Through these common mechanisms, both SARS-CoV-2 infection and social isolation can lead to mental health impairments [e.g., impaired memory, depression, psychoses, anxiety and posttraumatic stress disorder symptoms (PTSD)]. IL, Interleukin; TNF-α, tumor necrosis factor alpha; INF-γ, interferon gamma.

A further important aspect to be pointed out is the impact that the COVID-19 pandemic can have on people who are isolated to prevent the transmission of the virus and to prevent health system overload. Similar to possible mechanisms involved in the impacts of SARS-CoV-2 infection on mental health, social isolation may also be associated with dysfunctional psycho-neuroendocrine-immune interactions, which in turn can contribute to the development or the worsening of psychiatric disturbances (Figure 2). It urges to put all ours efforts in understanding the pathophysiology of COVID-19, including CNS infection and the risk of mental health compromise, but also the effects of this pandemic in the healthy isolated individuals, including children and adolescents, so that to prevent a “new generation” of groups in which the risk of developing mental disturbances, as anxiety or depression, could be increased. If nothing is done, we will probably be doomed to face a new mental health “pandemic” in the future.

In terms of social aspects, a number of short term simple attitudes or initiatives, can comprise the encouragement to: (a) strengthen bonds using social media and start thinking positively (140); (b) sleep properly and exercise regularly (141); (c) balance the diet, regular daily routine, relaxation exercise and other healthy lifestyle measures (142). On the other hand, people should be avoid: substance use, eating too much fast food, excessive online activity, excessive watching television, and believing fake news (142). It is also important to look for strategies that mitigate the impacts of COVID-19 on frontline healthcare providers. For instance, as recommended by Ho et al. (143), healthcare organizations should introduce shorter working periods, regular breaks, and rotating shifts. Individuals who experience moderate to severe and/or persistence distress should seek help from mental health professionals or in hospitals in cases of emergency situations (142). In addition, online consultation can be a potential alternative of delivering therapy (144).

We also believe that art (especially music) can be an ally in the quest to improving mental health, whether for inpatients, health care workers, or isolated people. A meta-analysis study reported that music can modulate cytokine levels (including reducing IL-6 levels), as well as neuroendocrine-immune responses triggered by stress, including physical stress caused by viral infection (145). In addition, it has been reinforced that music interferes positively in the immune system when subjected to acute stress (CO_2_ stress test), also regulating the function of IL-6 and the HPA axis (146). Therefore, music therapy can be a further relevant and simple strategy that might be adopted on a large-scale basis, for individuals in social isolation (also including medical staff).

Overall, it is important that political and health authorities pay attention to the mental health of infected and uninfected individuals during the pandemic, looking for prevention and treatment strategies, since poorer mental health can be associated with shorter life expectancy (147–149) and high economic burden (150, 151). Beyond the immediate and fundamental task of saving lives during SARS-CoV-2 pandemic, the due care of his mental health should be timely addressed. Protocols aiming at minimizing mental problems during the infection as well as during recovering after hospitalization must be designed. In addition, studies that evaluate the impact of isolation during SARS-CoV-2 pandemic on mental health are important as they can guide new strategies to preserve population mental health in other critical situations that we can live in the future.

Finally, it is noteworthy that the approach applied herein, related to psychoneuroimmunology in COVID-19, should be convergent with a social sciences approach so that to better understanding and to better tackling this disease. Hopefully, future studies may test the hypothesis outlined herein to better understand and consequently mitigate the impacts of COVID-19 on mental health.

## Data Availability Statement

The original contributions presented in the study are included in the article/Supplementary Material, further inquiries can be directed to the corresponding author.

## Author Contributions

ÍR, CF, and PO-S designed the study, managed literature searches, and wrote the manuscript. WS contributed to literature searches and writing of the manuscript. PP, EG, and WS critically reviewed the manuscript. All authors contributed to and have approved the final manuscript.

## Conflict of Interest

The authors declare that the research was conducted in the absence of any commercial or financial relationships that could be construed as a potential conflict of interest.
