# Supplementary material for: Psycho-Neuroendocrine-Immune Interactions in COVID-19: Potential Impacts on Mental Health
Source: Front Immunol. 2020 May 27;11:1170. doi: 10.3389/fimmu.2020.01170 (PMC7267025; doi:10.3389/fimmu.2020.01170)
Supplement: Supplementary file 1 [file Table_1.DOCX]

Supplementary Material

| Supplementary Table 1. Mental health outcomes in patients infected by MERS, SARS or COVID-19. | | | | |
| --- | --- | --- | --- | --- |
| Disease and References | **Sample size** | **Time after acute phase** | **Mental health outcomes** | **Factors associated with outcomes** |
| *MERS* |  |  |  |  |
| Kim et al (30) | n = 24 patients | During acute phase | 70.8% of MERS patients presented psychiatric symptoms (insomnia [29.2%], depressive mood [20.8%], tension [37.5%], disorientation [8.3%], impaired memory [8.3%], auditory hallucinations [8.3%] and aggressive outbursts [8.3%]). 41.7% of patients receive psychiatric diagnoses (adjustment disorders [12.5%], depressive disorders [8.3%], acute stress disorders [8.3%], anxiety disorders [8.3%] and mild neurocognitive disorder [4.2%] | ⋅⋅ |
| *SARS* |  |  |  |  |
| Cheng et al (28) | n = 10 patients with psychiatric complications (none of them had any psychiatric history) | During acute phase | Patients displayed anger, anxiety, suicidal ideas, depressive reaction (n = 7); hallucinatory and manic features (n = 3). 5 patients were diagnosed with adjustment disorder, 2 were diagnosed with organic hallucinosis, 2 with organic manic disorder and one had no diagnosis | Symptom severity of SARS, total isolation during treatment and steroid therapy |
| Chua et al (29) | n = 79 patients, of which 30 (39%) HCWs were infected  n = 145 healthy control subjects | During acute phase | SARS patients displayed increased stress levels compared to healthy control subjects (p < 0.04). In the SARS patient group, no significant differences were found between HCWs and non-HCWs | ⋅⋅ |
| Lee et al (31) | n = 1744 patients | During acute phase | 0.9% of patients were diagnosed with psychotic disorders (steroid-induced manic episode [n = 10]; steroid-induced psychotic disorder [n = 3]; major depressive episode with psychotic features [n = 1]; and psychotic disorder not otherwise speciﬁed [n=1]) | Family history of psychiatric illness and higher doses of steroid (steroid toxicity) |
| Lee et al (32) | n = 79 survivors (30 HCWs and 49 non-HCWs)  n = 96 survivors (33 HCWs and 63 non-HCWs) | Acute phase  1 year | SARS survivors displayed increased stress levels compared to matched community control subjects (p < 0.05). Stress levels were higher among females than those of male survivors (p < 0.05). In the SARS survivors, no significant differences were found between HCWs and non-HCWs  SARS survivors displayed increased stress levels compared to matched community control subjects (p = 0.001). Moderate-to-severe depressive symptoms (36.3%), severe depressive symptoms (4.4%), moderate-to-severe anxiety symptoms (36.7%) and severe anxiety symptoms (14.4%) were observed in SARS survivors. The proportion of participants with at least a moderate level of distress (> 2) on the 3 dimensions of posttraumatic symptoms was 32.2% (intrusion), 20.0% (avoidance), and 22.2% (hyperarousal). Stress levels were higher among females than those of male survivors (p < 0.05). In addition, women had higher scores on depression (p < 0.01), anxiety (p < 0.001), intrusion (p < 0.01), avoidance (p < 0.05) and hyperarousal (p < 0.05).  HCWs presented a high stress level (p < 0.001), more depressive and anxiety symptoms (p < 0.01, p < 0.001; respectively) compared to non-HCWs. In addition, HCWs displayed more PTSD symptoms compared to Non-HCW in 3 dimensions: intrusion (p<0.001), avoidance (p<0.05) and hyperarousal (p<0.05) | Females  Females and health care work were associated with worse mental health outcomes in the long-term |
| Sheng et al (33) | n = 102 patients | During acute phase  Convalescent phase | Patients reported insomnia (46.1%), impaired memory (41.2%), poor concentration (38.2%), low mood (36.3%), tension (36.3%), disoriented in time (33.3%) and place (3.9%), fear and panic (26.5%), pressured speech (20.6%), unstable mood (29.4%), euphoric mood (7.8%), pessimistic thinking (27.5%), overly optimistic (14.7%), social isolation (19.6%), reticent and wordless (25.5%), crying spell (23.5%), irritability/yelling (4.9%), persecutory ideas (3.9%), auditory hallucination (3.9%), visual hallucination (2.0%), abscondence (3.0%), noncompliance (3.0%) and suicidal idea (2.0%)  Patients reported insomnia (22.5%), impaired memory (43.1%), poor concentration (26.5%), low mood (18.6%), tension (20.6%), disoriented in time (7.8%) and place (3.0%), fear and panic (13.7%), pressured speech (11.8%), unstable mood (23.5%), euphoric mood (10.8%), pessimistic thinking (14.7%), overly optimistic (16.7%), social isolation (4.9%), reticent and wordless (7.8%), crying spell (13.7%), irritability/yelling (3.9%), persecutory ideas (2.0%), auditory hallucination (1.0%), abscondence (1⋅0%), noncompliance (2.9%), aggressive behaviors (1.0%) and self-injurious behaviors (1.0%) | Symptom severities of SARS and corticosteroids therapy were associated with symptoms of anxiety, depression, psychosis and behavioral problems. Health care work was associated with more cognitive problems  Effect of corticosteroids therapy persisted in the convalescent phase and was identiﬁed as a risk factor against psychological well-being. Health care work became a risk factor for anxiety-depression symptoms at convalescence |
| Wu et al (34) | n = 195 survivors | 1 month | 10% to 18% reported symptoms related to PTSD, anxiety and depression | High perceived life threat and low emotional support was associated symptom severity; female and low education levels was associated symptoms of avoidance; and knowing someone who had SARS was associated depressive symptoms |
| Hong et al (35) | n = 70 patients  n = 61 patients  n = 57 patients  n = 58 patients  n = 57 patients | 53 days  7 months  10 months  20 months  46 months | 40% of SARS survivors presenting PTSD symptoms  41% of SARS survivors presenting PTSD symptoms  38⋅6% of SARS survivors presenting PTSD symptoms  39⋅7% of SARS survivors presenting PTSD symptoms  42⋅1% of SARS survivors presenting PTSD symptoms | Higher number of female survivors in the sample |
| Lam et al (36) | n = 181 survivors (6 with history of psychiatric disorders before contracting SARS) | 31-51 months | The most common diagnoses were PTSD (23.2%), depression (16.6%), somatoform pain disorder (15.5%), panic disorder (13.8%), obsessive compulsive disorder (6.6%) | Health care work during the SARS epidemic, being unemployed (including being a housewife or retired) at follow-up, having the perception of social stigmatization, and having applied to the SARS survivors' fund were associated with an increased risk of psychiatric morbidities at follow-up.Chronic fatigue was associated with active psychiatric illness |
| Mak et al (37) | n = 90 patients (6.7% with pre-SARS psychiatric disorder) | Up to 30 months | Post-SARS cumulative incidence: major depression (13.3%), dysthymia (2.2%), PTSD (25.6%), panic disorder (7.8%), agoraphobia (3.3%), social phobia (1.1%), generalized anxiety disorder (3.3%), post-SARS psychotic symptoms (0%) and alcohol- or substance-related disorders (0%).  30 months post-SARS: major depression (44.4%), dysthymia (2.2%), PTSD (47.8%), panic disorder (13.3%), agoraphobia (6.6%), social phobia (1.1%), post-SARS psychotic symptoms (4.4%) and alcohol- or substance-related disorders (0%)  Recovery rate: major depression (31.1%), dysthymia (0%), PTSD (22⋅2%), panic disorder (5.6%), agoraphobia (3.3%), social phobia (0%), post-SARS psychotic symptoms (4.4%) and alcohol- or substance-related disorders (0%) | ⋅⋅ |
| Moldofsky and Patcai (38) | n = 22 survivors (19 females and 3 males; all except one being HCW)  n = 21 drug free female patients, who fulfilled criteria for fibromyalgia  n = 8 healthy females | 13 to 36 months | Post-SARS subjects displayed more mild to moderate depressive symptoms (p < 0.0001), more sleep disturbances (p < 0.0001), more fatigue post-sleep (p < 0.05) and more myalgia pre- and post-sleep (p < 0.01) compared to healthy control subjects. 2 patients showed symptoms suggestive of PTSD | ⋅⋅ |
| *COVID-19* |  |  |  |  |
| Bo et al (4) | n = 714 clinically stable patients | During acute phase | The prevalence of self-reported significant PTSD symptoms was 96.2% | ⋅⋅ |
| Kong et al (5) | n = 144 patients | During acute phase | 34.72% of patients presented anxiety symptoms (17.36%, 12.5% and 4.86% appeared to have mild, moderate and severe anxiety levels, respectively), and 28.47% displayed depression symptoms (13.89%, 10.42% and 4.17% appeared to have mild, moderate and severe depression levels) | Less social support, gender (female), older age and lower oxygen saturation were associated with anxiety. In addition, social support, older age and family infection with SARS-CoV-2 were associated with depression |
|  |  |  |  |  |

MERS = Middle East Respiratory Syndrome; SARS = Severe Acute Respiratory Syndrome; COVID-19 = Coronaviruses Disease 2019; CoV = Coronavirus; HCWs = Healthy Care Workers; PTSD = Post-traumatic stress disorder.
